# Supplementary material for: Interferon Regulatory Factor 3-Mediated Signaling Limits Middle-East Respiratory Syndrome (MERS) Coronavirus Propagation in Cells from an Insectivorous Bat
Source: Viruses. 2019 Feb 13;11(2):152. doi: 10.3390/v11020152 (PMC6410008; doi:10.3390/v11020152)
Supplement: Supplementary file 1 [file viruses-11-00152-s001.pdf]

Supplementary

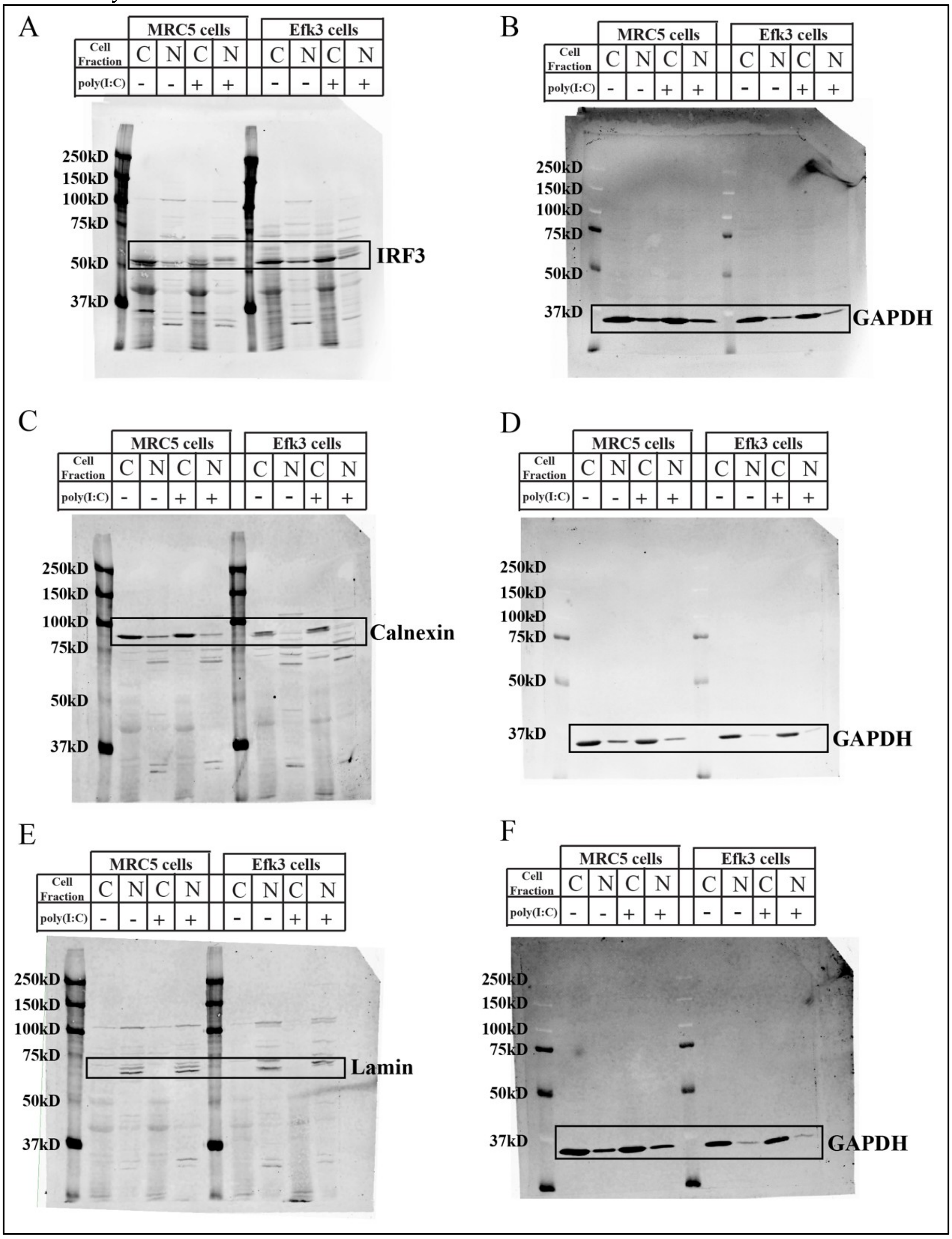

Figure S1. Full size blots for Figure 3C.

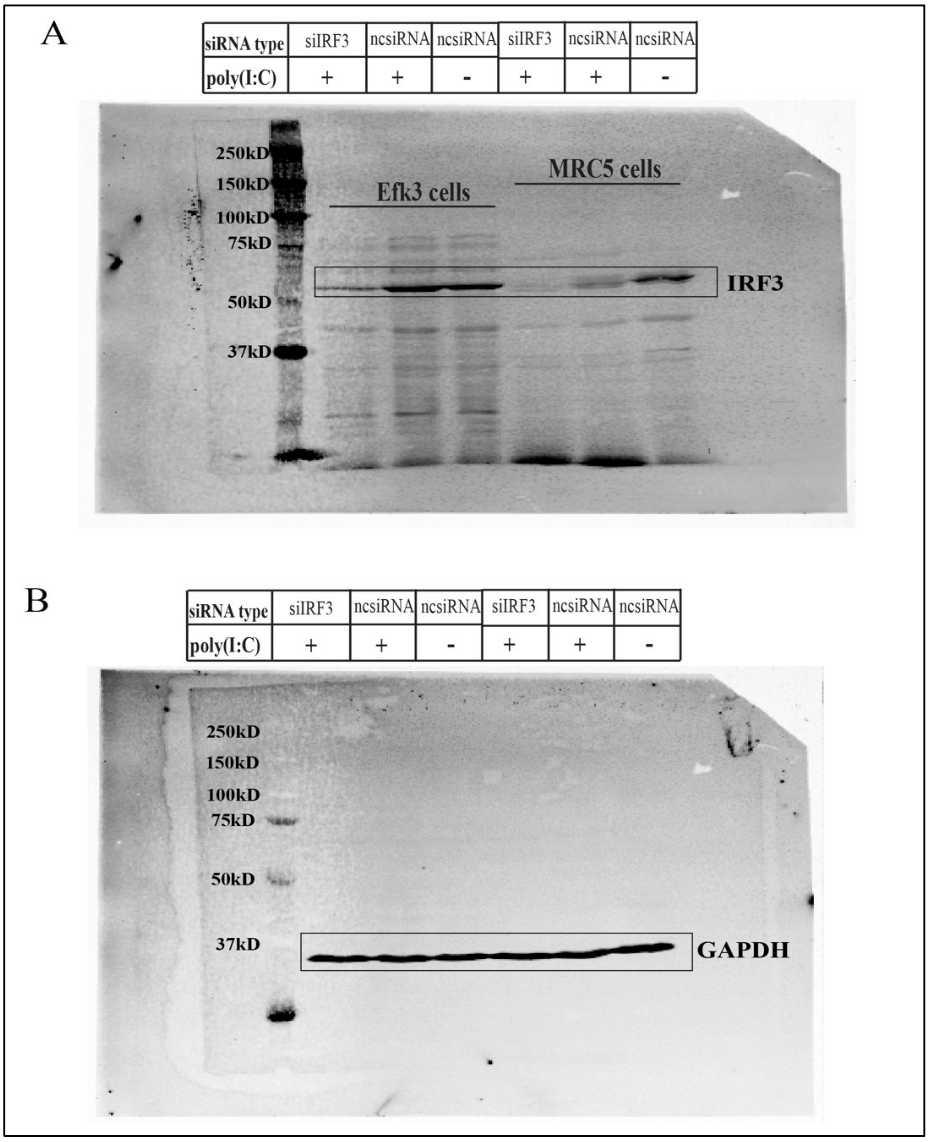

Figure S2. Full size blots for Figures 4A and 4B.

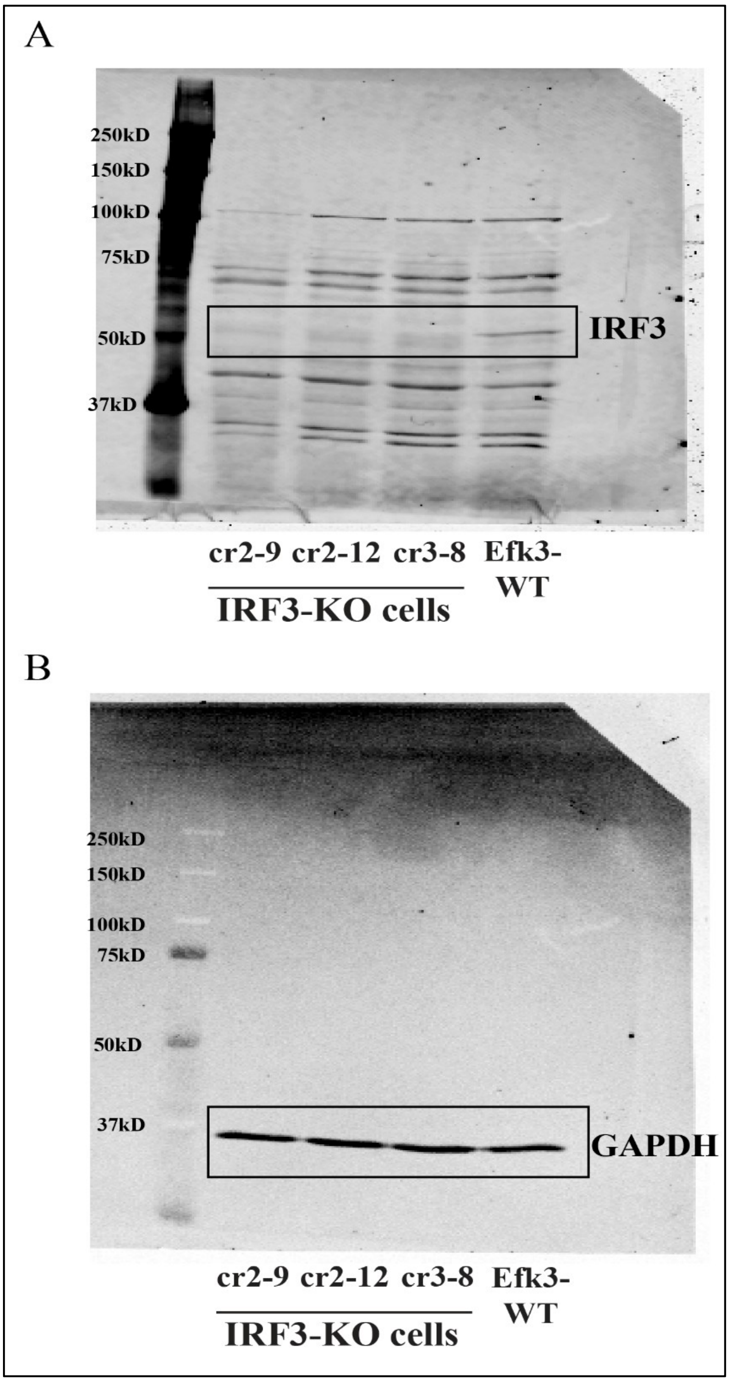

Figure S3. Full size blots for Figure 4D.

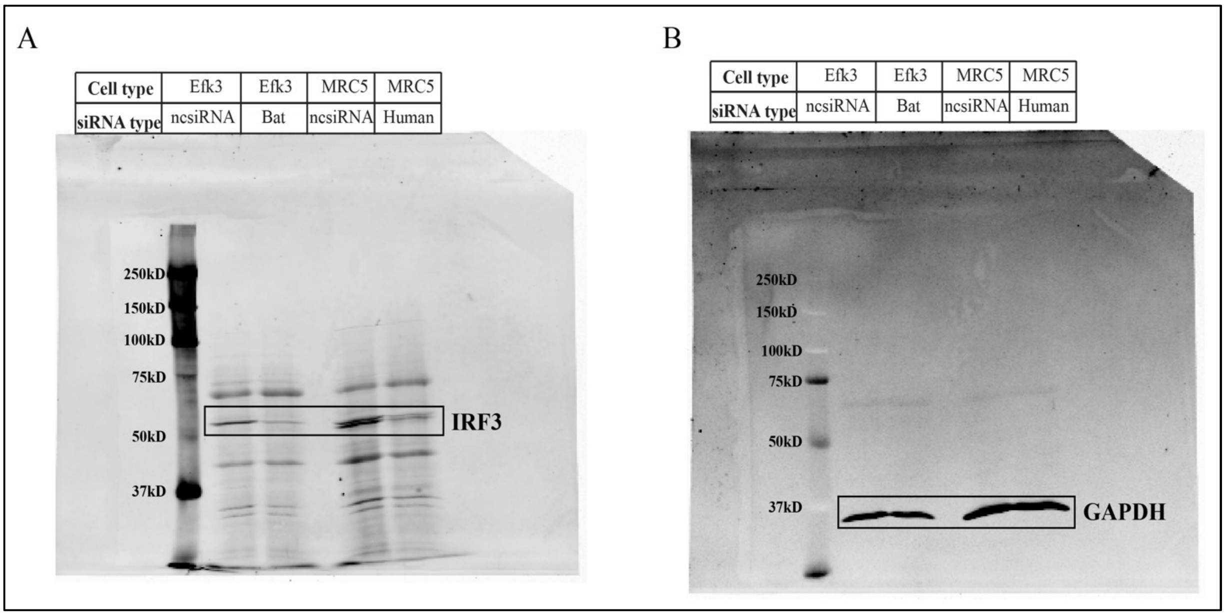

Figure S4. Full size blots for Figure 5A.
